# Psychometric properties of the Chinese version of the Functional Assessment of Cancer Therapy-Cervix (FACT-Cx) measuring health-related quality of life

**DOI:** 10.1186/1477-7525-10-124

**Published:** 2012-10-03

**Authors:** Yan Ding, Yan Hu, Ingalill R Hallberg

**Affiliations:** 1 Nursing Department, Obstetrics and Gynecology Hospital of Fudan University, No. 419 Fang Xie Road, Shanghai, 200011, China; 2 Nursing School, Fudan University, No. 305 Feng Lin Road, Shanghai, 200032, China; 3 Department of Health Sciences, Lund University, BaravÃ¤gen 3, Lund, SE-22100, Sweden

**Keywords:** Health-related quality of life, FACT-Cx, FACT-G, Chinese version, Psychometric properties, Cervical cancer

## Abstract

**Background:**

The Functional Assessment of Cancer Therapy (FACT) is one of the most commonly used self-report instruments for evaluation of health-related quality of life in oncology patients. However, cultural considerations necessitate testing of the subscales in different populations. We sought to qualitatively and quantitatively investigate the applicability and psychometric properties of the Chinese version of the FACT-Cervix (FACT-Cx) in Chinese women with cervical cancer.

**Methods:**

Ten personal interviews were conducted in order to explore patients’ opinions about the scale and its items in depth. In addition the questionnaire was administered to 400 women with cervical cancer to test its psychometric properties. Reliability was assessed using Cronbach’s alpha coefficient and item-subscale correlation while validity was evaluated using factor analysis and known-group validity.

**Results:**

Some items related to sex and the ability to give birth were questioned in the personal interviews, mostly regarding their significance and acceptance in the Chinese cultural context. The Cronbach’s alphas of FACT-Cx and the subscales were greater than 0.7, except for the cervical-cancer-specific subscale which was 0.57. Factor analysis demonstrated that the FACT-G construct generally paralleled the original. There were significant differences in the FACT-Cx and some subscales between those receiving and not receiving treatment and among the patients with different performance status.

**Conclusions:**

In general, psychometric properties of the Chinese version supported its use with cervical cancer patients in Mainland China. Further work is needed to improve the psychometric adequacy of the cervical-cancer-specific subscale and adjust it to cultural considerations.

## Background

Cervical cancer is the second most common cancer among women worldwide. About 83% of cases occur in developing countries, such as China and India, representing 15% of women’s cancers [1]. Current Chinese estimates indicate that cervical cancer is the 5th most frequent cancer among women aged 15–44 years [1]. The increased survival rate due to medical developments means that women with cervical cancer will probably have a life expectancy of more than twenty years after diagnosis and treatment. Having to live with cervical cancer and the outcomes of the treatment, how the women can reclaim or keep their quality of life are increasingly becoming as important as how to prolong survival. In addition to morbidity and the 5-year survival rate, health-related quality of life (HRQOL) has been regarded as a key outcome indicator in oncology.

According to Cella et al., HRQOL has two fundamental components: multidimensionality and subjectivity. The multidimensionality of HRQOL refers to the broadness of areas that patients regard as important for their lives, including physical, functional, emotional, and social well-being. Subjectivity refers to the fact that HRQOL can only be understood from the patient’s perspective [2]. Based on this, Cella and his colleagues developed an instrument to empirically measure HRQOL, the Functional Assessment of Cancer Therapy (FACT). To date the FACT is one of the most commonly used self-reported instruments for evaluation of HRQOL in oncology patients. The instrument system has been translated into over 60 languages, including Chinese, by the organization that developed the FACT [3,4]. The FACT-general (FACT-G) is the core scale of the instrument system and consists of four dimensions measuring physical well-being (PWB), social/family well-being (SFWB), emotional well-being (EWB), and functional well-being (FWB). To evaluate precisely and responsively the HRQOL of patients with a specific cancer, some cancer-site-specific subscales have also been developed to address specific concerns related to that particular cancer, such as the lung-cancer-specific subscale, breast-cancer-specific subscale, and cervical-cancer-specific subscale (CCS). Assessing the HRQOL of patients with cervical cancer requires a combination of the FACT-G and CCS, which constitutes the full scale, the FACT-Cervix (FACT-Cx).

The FACT-G and some cancer-site-specific subscales, developed in western contexts and applied to populations in western countries, have demonstrated adequate reliability, validity and sensitivity to change, and are thus valid and reliable choice for HRQOL measurement [3-7]. To date reliability and validity data for gynecologic cancer-specific scales are available only for the FACT-Ovarian (FACT-O) and FACT-Vulva (FACT-V). Cronbach’s alpha of the FACT-O was 0.92 and test-retest reliability was 0.81 [8]. Cronbach’s alpha of the FACT-V was 0.92 [7]. To prove known-group validation, a significant difference was found in the FACT-V and Vulva subscale among the subgroups with different performance status and disease stages [7]. Similarly, the total FACT-O and emotional well-being scores could distinguish subgroups of patients with different performance status, as well as patients under and not under active treatment [8]. To the best of our knowledge there are no published studies on psychometric tests of the FACT-Cx or CCS.

Despite these results, the validity and reliability of an instrument, when applied to a population in a different cultural context, may differ from those of the original. Because there is agreement that HRQOL is substantially influenced by culture, it is inappropriate to apply the FACT, which was developed in a western cultural context, directly to a Chinese population. This is particularly so given some unique health-related philosophies in Chinese culture. For example, in traditional Chinese medicine, health is viewed as harmony between the forces of Yin and Yang within and between the body and its environment. Illness, by contrast, is viewed as an imbalance or disequilibrium of these powerful forces. Similarly a satisfying social life means being in harmony with one’s social environment [9]. Through this means one can acquire a good quality of life. Due to the different cultural background, the cultural equivalence and psychometric properties of the FACT-G and FACT-Cx should first be evaluated.

Although the FACT has been applied to Chinese cancer patients, few studies have reported on its reliability and validity, particularly those from Mainland China [10]. The FACT-G was primarily tested for its psychometric properties in cancer patients in Hong Kong. This special Chinese administrative region previously under colonial government by the United Kingdom is a blend of western and Chinese cultures. It was concluded that although more work was needed to increase its adequacy, the translated scale could reasonably be used for Chinese populations in clinical settings [11]. Later Wan et al. evaluated the FACT-G in 552 cancer patients in Mainland China. Their study demonstrated acceptable validity and reliability [12]. However, few studies have evaluated the site-specific subscale together with the FACT-G as applied in a Mainland Chinese context. To date we found that only the FACT-Lung and FACT-Breast were evaluated for psychometric properties in patients with lung and breast cancer in Mainland China [13,14]. In summary, while the FACT-Cx has been translated into Chinese by the FACT development organization, no investigation of its psychometric properties has to our knowledge been carried out in Mainland China.

The purpose of this study was to qualitatively and quantitatively investigate the applicability, validity and reliability of the Chinese version of the FACT-Cx in women with cervical cancer in Mainland China.

## Methods

### Design

The study combined quantitative and qualitative methods in order to gain insight into the suitability of the Chinese version of the FACT-Cx for use with women with cervical cancer in Mainland China. We used a survey to test the psychometric properties of the scale. However, to ensure applicability we also carried out personal interviews in a smaller sample. This allowed us to discover whether participants found any of the items difficult to respond to, which could lead to a large internal drop-out. Thus face-to-face personal interviews complemented the quantitative method and provided detailed information about items regarded as problematic or difficult to respond to.

### Subjects

The subjects were recruited in two specialized university hospitals in Shanghai, China. Their reputation regarding gynecological cancer treatment attracted patients with cervical cancer not only from Shanghai but from all over China. Thus the cervical cancer population in the two hospitals presented a rich sample pool for the study. Both ethics committees of the two hospitals approved the study (approval numbers 2008*–*40 and 090472–12).

Patients diagnosed with cervical cancer were considered potential subjects. The inclusion criteria were: (1) pathologically diagnosed with cervical cancer; (2) Chinese resident with full knowledge of the Chinese language; (3) not diagnosed with any other type of cancer; (4) not diagnosed with a major medical or psychiatric condition. Informed consent was obtained from each potential subject prior to data collection.

### Measure to be tested: the Chinese version of the FACT-Cx

FACT-Cx comprises FACT-G and CCS. The FACT-G has four domains, seven items measuring PWB, seven items measuring SFWB, six items measuring EWB, and seven items measuring FWB. The CCS, also called “additional concerns”, contains 15 additional items specifically related to cervical cancer and its treatment sequelae. The FACT-Cx can be self-administered or used in an interview format. Patients are asked to rate from 0 to 4 how they feel today and have felt during the preceding 7 days. There are some reverse-stated items, which have to be negatively scored. The score for each domain is obtained by adding up all the item scores within that domain. The FACT-G score is the summed score for the four domains. The FACT-Cx score is the total score for the FACT-G plus CCS (Table 1). A higher score indicates a better HRQOL. The possible range of scores is 0 to 168 [15].

**Table 1:** Scoring method and Cronbach’s alpha coefficients of the FACT-Cx

**Subscales**	**Items**	**Score range**	**Cronbach’s alpha**
Physical wellbeing (PWB)	7	0-28	0.736
Social/family wellbeing (SFWB)	7	0-28	0.817
Emotional wellbeing (EWB)	6	0-24	0.759
Functional wellbeing (FWB)	7	0-28	0.807
FACT-G (PWB+SFWB+EWB+FWB)	27	0-108	0.872
Cervical–cancer-specific subscale (CCS)	15	0-60	0.570
FACT-Cx (FACT-Cx + CCS)	42	0-168	0.876

The Chinese version was provided by the Center on Outcome, Research and Education (CORE), Evanston Northwestern Healthcare, Northwestern University, which is mainly involved in the development, translation and linguistic validation of FACT measures. To our knowledge it has not been tested before in Mainland China [16].

### Data collection

#### Quantitative data collection

Consecutive sampling was used for quantitative data collection. Four hundred respondents for the questionnaire survey were recruited simultaneously from outpatient and inpatient settings. There was an on-site coordinator in each setting who informed the investigator when there were patients who met the inclusion criteria. The investigator approached the patients seeking informed consent. Thereafter the patients were asked to complete the Chinese version of the FACT-Cx in the presence of the investigator. Once a subject had finished the questionnaire, the investigator checked that it was complete and marked those items that the subject had not wanted to respond to.

#### Qualitative data collection

Consecutive sampling was also used for qualitative data collection. The first author chose the ten subjects and carried out all the personal interviews. Patients from the sample for the questionnaire survey were approached and asked to participate in the interviews if they were experienced as open, talkative, and eloquent by the first author. Oral explanation of the study was provided and confidentiality was assured. It was also stressed that participation was voluntary and that they could withdraw from the study at any time without giving any reason. Because the first two patients both did not agree to tape recording this was abandoned. Once consent was given, a patient was asked to complete the questionnaire by herself. Once completed it was checked by the interviewer for missing data or other problems. The interviewer then asked about each FACT-Cx item as follows: whether it was clear and easy to understand; whether there was a better way to express the idea or if there were any related comments; whether there were any items that were difficult to understand, irrelevant or offensive; whether the patient had any other comments to make in general. The patients were encouraged to describe any reaction or reflection in relation to each item and further questions were asked if needed to obtain further clarification about their views. Finally the notes were shown to the patient for validation. Each interview took around 1.5 to 2.5 hours. The presence of family members during the interviews was discouraged to avoid any influence on patients’ responses.

In addition, a form was developed to collect performance status and demographic and socio-economic characteristics. Performance status was divided into five categories: normal activity, without symptoms; some symptoms but not requiring bed rest during daytime; requiring bed rest for less than 50% of daytime; requiring bed rest for more than 50% of daytime; and unable to get out of bed. All the subjects were asked to fill in the form. Their disease characteristics were obtained from their medical records, such as pathological diagnosis, disease stage, and treatment modalities.

### Data analysis

The item-subscale correlation and Cronbach’s alpha coefficient were calculated to evaluate internal consistency. Higher than 0.2 for the item-subscale correlation and higher than 0.7 for the alpha coefficient were regarded as acceptable [17,18]. Validity was assessed in terms of construct validity and known-group validity. Based on the operational definition of HRQOL, the four subscales of the FACT-G were supposed to be interrelated. Thus an oblique rotation method for factor analysis was performed in this study, forcing a four-factor solution to replicate the original work [3,6,19]. Each item was expected to load in its original domain with the largest factor loading. The Eigenvalues of the four factors should be greater than 1 [19]. To evaluate known-group validity, the mean scores of the FACT-Cx, FACT-G and the subscales were compared with Student’s *t*-test between the two groups under active treatment and not under active treatment. The Kruskal-Wallis test was used to compare the scores of the FACT-Cx, FACT-G and the subscales among the five groups categorized by the levels of performance status, as well as among the four groups at varying disease stages. This was because some groups were too small and not equal in size to the other groups [20]. It was hypothesized that the HRQOL questionnaire could differentiate the patients under active treatment from those who were not, as well as patients at various stages or with varying levels of performance status. The known-group validity was regarded as acceptable if there were statistically significant differences in HRQOL. To compare the responses to item C6 (I have concerns about my ability to have children) between the women with and without children, the Mann–Whitney *U*-test was employed because the shape of the sample distributions was not normal [21]. Statistical analyses were performed using the Statistical Package for Social Science 13.0 (SPSS13.0). All-available approach was defaulted to manage the missing data, which means using all valid values for each variable and only ignoring the missing values [22].

## Results

### Personal interviews

The ten patients ranged from 30 to 47 years of age. Eight were diagnosed with squamous cell carcinoma and two with adenocarcinoma. Cancer stage varied from Ib to IIa. Six patients were under active treatment and all of them had borne a child before being diagnosed with cervical cancer (Table 2).

**Table 2:** Characteristics of the subjects

**Characteristics**	**Qualitative interview N(10)**	**%**	**Quantitative survey N(400)**	**%**
**Age, y**				
Range	30∼47		19∼68	
Mean±SD	35.50±5.36		42.63±8.10	
**Education background**				
Primary school	1	10	87	21.8
Secondary school	2	20	144	36.0
High school	4	40	111	27.8
College level or above	3	30	56	14.0
Missing			2	0.5
**Marital status**				
Married	9	90	378	94.5
Unmarried			3	0.8
widowed			3	0.8
Divorced	1	10	15	3.8
Separated			1	0.3
**Family income (RMB/month)**				
<2000	2	20	121	30.3
2000∼5000	6	60	178	44.5
5001∼8000			39	9.8
8001∼10000	1	10	20	5.0
>10000	1	10	34	8.5
Missing			8	2.0
**Perceived financial status**				
Rich			18	4.5
Average	2	20	304	76.0
Poor	8	80	78	19.5
**Type of medical payment**				
full insurance			34	8.5
=>50% insurance	6	60	132	33.0
>50% self-paid	1	10	108	27.0
Fully self-paid	3	30	119	29.8
Missing			7	1.8
**Pathological diagnosis**				
squamous cell carcinoma	8	80	344	86.0
adenocarcinoma	2	20	30	7.5
mixed			14	3.5
Missing			12	3.0
**Stage**				
0			51	12.8
I	8	80	245	61.3
II	2	20	86	21.5
III			3	0.8
Missing			15	3.8
**Treatment modality**				
No treatment	4	40	156	39.0
Surgery	4	40	164	41.0
Chemotherapy			41	10.3
Radiotherapy			6	1.5
Surgery and chemotherapy	2	20	23	5.8
Radiotherapy and chemotherapy			3	.8
Surgery and Radiotherapy and chemotherapy			5	1.3
Missing			2	.5
**Performance status**				
Normal activity, without symptoms	6	60	189	47.3
Some symptoms, but does not require bed rest during daytime	4	40	81	20.3
Requires bed rest for less than 50% of daytime			28	7.0
Requires bed rest for more than 50% of daytime			90	22.5
Unable to get out of bed			8	2.0
Missing			4	1.1
**Having children or not**				
Yes	10	100	373	93.3
Not			25	6.2
Missing			2	.5

Abbreviation: RMB, Ren-Ming-Bi (Chinese dollar. One RMB roughly equal to .15 US dollars).

All the subjects filled in the questionnaire independently in 10–20 minutes. No item was regarded as difficult to understand. One patient mentioned some other things that should be included however these were already in the questionnaire. Five items were questioned and regarded as inappropriate, one from the SFWB subscale, item S7 “I am satisfied with my sex life”; the others all from the CCS, item C4 “I feel sexually attractive”; item C5 “My vagina feels too narrow or short”; item C6 “I have concerns about my ability to have children”; and item C8 “I am interested in sex” (Table 3).

**Table 3:** Subjects’ item responses in the qualitative and quantitative investigation

**Item**	**Personal interviews (n=10)**	**Questionnaire survey (n=400)**
	**Comments**	**Number of non-response (%)**
**In the social/family wellbeing subscale (SFWB):**		
S7: I am satisfied with my sex life	Too private to answer (n=1)	97 (24.25)
**In the cervical-cancer-specific subscale (CCS):**		
C3: I am afraid to have sex	No comment	15 (3.75)
C4: I feel sexually attractive	Too private to answer (n=1)	25 (6.26)
C5: My vagina feels too narrow or short	It seems to be a question for sexual partner (n=1)	18 (4.50)
C6: I have concerns about my ability to have children	Little significant due to uterus removal (n=1);	21 (5.25)
	Have a child already, so the question is of little significance (n=2);	
	The question is conditional on having or not having a child (n=1).	
C8: I am interested in sex	Too private to answer (n=2)	23 (5.75)

### Survey

The 400 subjects ranged in age from 19 to 68 years with a mean age of 42.63 years; 86.0% of them were diagnosed with squamous cell carcinoma; Time since diagnosis ranged from 0 to 79 months with a mean of 5.51; 61.3% were in stage I; 61% were under active treatment and 93.3% had borne a child before being diagnosed with cervical cancer. Although 74.8% of the subjects rated their family income lower than 5000 RMB per month (One RMB is roughly equivalent to 0.15 US dollars), 76.0% perceived their family financial status as average, only 4.5% rated themselves as poor. Of the patients 56.8% had to pay more than half their medical expenses themselves and 20.8% perceived their financial burden as “extreme” (Table 2).

As in the personal interviews, some patients declined to answer the sex-related items. For S7 (I am satisfied with my sex life), the non-response rate was as high as 24.25% (Table 3).

All the items in the FACT-G had item-subscale correlations higher than 0.50 except for item E2 which was 0.34. In the CCS subscale, the item-subscale correlations ranged from 0.18 to 0.49. Item C6 (I have concerns about my ability to have children) alone was lower than 0.2. The Cronbach’s alpha coefficients of the FACT-Cx and all the subscales were higher than 0.7 except for the CCS subscale which was 0.57 (Table 1). Dropping item C6 slightly increased the alpha coefficient to 0.58 for the remaining 14 items in the CCS. However dropping more items could not elevate the alpha coefficients further.

In factor analysis, the forced four-factor solution accounted for almost half of the explained variance (49.84%). The Eigenvalues of all the four principal components were greater than 1. The first principal component was comparable to EWB; the second component to SFWB; the third to PWB, and the fourth to FWB. However, item P6 in PWB cross-loaded on the first and third principal components. Item E2 in EWB cross-loaded strongly on the second, third and forth principal components rather than on the first principal component (Table 4).

**Table 4:** Items in each original subscale of the FACT-G in relation to factor structure and loadings of this study

**Factor structure originally**	**Component in this analysis**				**Com.**
	**1**	**2**	**3**	**4**	
**Physical wellbeing (PWB):**					
P1: I have a lack of energy	-.042	-.014	**.551**^**a**^	-.091	.321
P2: I have nausea	-.011	.029	**.611**^**a**^	.007	.371
P3: Because of my physical condition, I have trouble meeting the needs of my family	.173	.073	**.520**^**a**^	-.117	.422
P4: I have pain	.010	.067	**.696**^**a**^	.014	.493
P5: I am bothered by the side effects of treatment	.264	.225	**.402**^**a**^	.051	.337
P6: I feel ill	**.476**^**a**^	-.132	.302	-.142	.468
P7: I am forced to spend time in bed	.010	-.047	**.755**^**a**^	-.064	.598
**Social/family wellbeing (SFWB):**					
S1: I feel close to my friends	-.108	**.665**^**a**^	.029	-.082	.471
S2: I get emotional support from my family	.136	**.767**^**a**^	.054	.148	.580
S3: I get support from my friends	-.117	**.663**^**a**^	.027	-.130	.493
S4: My family has accepted my illness	-.093	**.704**^**a**^	-.012	-.007	.489
S5: I am satisfied with family communication about my illness	-.040	**.814**^**a**^	-.023	.073	.627
S6: I feel close to my partner (or the person who is my main support)	.138	**.745**^**a**^	-.048	.018	.587
S7: I am satisfied with my sex life	-.019	**.500**^**a**^	.125	-.074	.300
**Emotional wellbeing (EWB):**					
E1: I feel sad	**.619**^**a**^	.073	.123	-.141	.555
E2: I am satisfied with how I am coping with my illness	.038	**.285**^**a**^	-.277	-.238	.223
E3: I am losing hope in the fight against my illness	**.561**^**a**^	.137	.047	.018	.361
E4: I feel nervous	**.827**^**a**^	-.070	-.090	-.083	.696
E5: I worry about dying	**.852**^**a**^	-.109	-.030	-.069	.745
E6: I worry that my condition will get worse	**.912**^**a**^	-.101	-.014	.017	.801
**Functional wellbeing (FWB):**					
F1: I am able to work (include house-work)	-.182	-.072	.312	**-.721**^**a**^	.601
F2: My work (include house.work) is fulfilling	-.008	-.066	-.071	**-.804**^**a**^	.594
F3: I am able to enjoy life	.115	.034	-.102	**-.759**^**a**^	.641
F4: I have accepted my illness	.145	.234	-.108	**-.479**^**a**^	.413
F5: I am sleeping well	.096	.017	.105	**-.389**^**a**^	.229
F6: I am enjoying the things I usually do for fun	.155	.007	.097	**-.562**^**a**^	.451
F7: I am content with the quality of my life right now	.131	.153	.078	**-.626**^**a**^	.593
Eigenvalues	6.552	3.401	1.948	1.556	
Variance contribution (%) 49.842	24.268	12.597	7.215	5.762	

^**a**^ The greatest factor loading for each item.

Abbreviation: Com, Communalities.

There was a significant difference in the PWB, CCS, FACT-G, and FACT-Cx between the two groups under treatment and not under treatment (Table 5). There was also a significant difference in the PWB, FWB, CCS, FACT-G and FACT-Cx among the five groups with different levels of performance status (Table 6). Among the four groups categorized by disease stage, no statistically significant difference was found in the FACT-Cx and the subscales, except for SFWB (Table 7).

**Table 5:** Comparison of HRQOL between the groups receiving and not receiving treatment

	**Receiving treatment (n=242)**	**Not receiving treatment (n=156)**	**t**	**p-value**
	**Mean (SD)**	**Mean(SD)**		
Physical wellbeing (PWB)	20.03(5.05)	23.23(3.65)	−7.156	<.001**
Social/family wellbeing (SFWB)	22.11(5.34)	23.19(4.34)	−0.772	.440
Emotional wellbeing (EWB)	17.67(4.92)	17.48(5.11)	0.364	.716
Functional wellbeing (FWB)	16.94(5.98)	17.02(6.22)	−0.117	.907
FACT-G	76.18(15.79)	80.40(13.92)	−2.139	.033*
Cervical-cancer-specific subscale (CCS)	41.95(6.55)	43.50(5.57)	−2.168	.031*
FACT-Cx	117.46(20.31)	124.47(17.44)	−2.663	.008**

* P<.05; ** P<.01.

**Table 6:** Comparison of HRQOL among the groups by performance status

**Performance Status**	**0 (n=189)**	**1 (n=81)**	**2 (n=28)**	**3 (n=90)**	**4 (n=8)**	**Chi-Square**	**P value**
	**Mean (SD)**	**Mean (SD)**	**Mean (SD)**	**Mean (SD)**	**Mean (SD)**		
PWB	23.47(3.58)	20.68(4.30)	20.00(4.02)	17.11(5.11)	16.63(1.89)	110.741	<.001**
SFWB	19.70(4.64)	20.37(4.19)	19.89(4.61)	20.71(3.80)	19.25(4.89)	3.487	.480
EWB	18.07(4.57)	16.62(5.28)	17.64(5.45)	17.44(5.09)	15.88(6.92)	4.620	.329
FWB	18.23(6.39)	16.64(5.24)	15.50(4.00)	15.32(5.78)	11.38(6.80)	22.738	<.001**
FACT-G	79.51(13.25)	74.30(14.22)	73.04(13.10)	70.56(14.45)	63.13(14.89)	30.392	<.001**
CCS	43.53(5.69)	40.99(6.64)	42.56(5.77)	41.77(6.84)	37.75(5.06)	12.535	.014*
FACT-Cx	122.36(17.13)	115.14(18.88)	114.60(15.65)	111.37(19.18)	100.88(18.87)	23.927	<.001**

* P<.05; ** P<.01.

**Table 7:** Comparison of HRQOL among the groups by stage

**Stage**	**0 (n=51)**	**I (n=245)**	**II (n=86)**	**III (n=3)**	**Chi-Square**	**P value**
	**Mean (SD)**	**Mean (SD)**	**Mean (SD)**	**Mean (SD)**		
PWB	22.47(4.40)	20.86(5.01)	21.19(4.56)	20.33(2.31)	5.329	.149
SFWB	18.78(5.07)	19.82(4.44)	21.48(3.46)	19.33(6.43)	14.588	.002**
EWB	17.88(4.43)	17.83(4.85)	16.93(5.56)	20.67(3.21)	2.643	.450
FWB	18.63(5.70)	16.60(6.32)	17.34(5.51)	12.67(3.79)	7.234	.065
FACT-G	78.09(13.55)	75.06(14.84)	76.82(13.77)	73.00(1.73)	2.530	.470
CCS	44.05(5.92)	42.37(6.53)	42.22(5.53)	36.00(13.45)	3.585	.310
FACT-Cx	121.55(17.22)	116.53(19.78)	118.55(16.99)	109.00(14.73)	2.798	.424

** P<.01.

Twenty-five women had not borne a child. Comparing the scores for item C6 (I have concerns about my ability to have children) between the women with a child and those without, using Mann–Whitney *U*-test, the value of Z was −4.248 and p-value was lower than 0.001, suggesting that those who had not given birth had concerns.

## Discussion

This study is to the best of our knowledge the first to assess the psychometric properties of the Chinese version of the FACT-Cx in Mainland China. It integrated quantitative and qualitative methods to place the statistical results in context, with the qualitative data adding depth and meaning to the quantitative results [23].

The personal interviews demonstrate that the Chinese version of the FACT-Cx, in terms of its understandability and administration, has acceptable feasibility. They however provide some insights into the distinct perspectives of Chinese women with cervical cancer concerning their reproductive ability. They were much less concerned about the loss of their reproductive ability than subjects in similar studies in the western world. The reason may be the “One Child Policy”, which has long been in force in Mainland China limiting Han ethnic Chinese couples to one child only. Most women have already borne a child before being diagnosed with cervical cancer, and thereby regardless of whether or not they were able to reproduce, they could not have another baby. Given these circumstances, it makes sense that item C6 “I have concerns about my ability to have children” appeared to have little significance for women in Mainland China, even though treatment for cervical cancer deprived their ability to have children. This differs from the views about loss of childbearing ability in western women with cervical cancer. Schover summarized the literature about the effects of cervical cancer treatment on sexuality and found that reproductive concerns were a major issue for survivors of cervical cancer. Their mental suffering arising from the loss of childbearing is substantial [24-26]. However, it is worth noting that all the ten women interviewed were one-child mother. In the following quantitative investigation, there was a significant difference in the score for the item related to reproductive ability between those with a child and those without. This indicated that the women without a child were concerned about their reproductive ability. Thus the findings apparently reflect a political decision and might change if the policy were changed.

The interviews also revealed that Chinese women tend to regard sexual issues as private. This was confirmed by the relatively low item response rates for sex-related items in the quantitative study. Chinese culture retains conservative values concerning sexual issues and is generally less sexually outspoken than western culture. Taoism, one major Chinese philosophy, suggests the regulation of sexual activities to preserve one’s health [27]. A manifestation of this is the Chinese cultural belief that intimacy between close partners is more independent of sexual relations than it is in the West [11]. Moreover, influenced by Chinese tradition in the long term, active sex is regarded by many people as the reason for the occurrence and progression of gynecological cancer [28]. Thus patients are reluctant to talk to strangers about the “sensitive” topics such as sexuality. This may be the main underlying factor affecting their responses to sex-related items [11]. Nevertheless, a caveat for clinical professionals providing healthcare for cervical cancer survivors in a Chinese context is in order. The hesitation to openly discuss the sexual problems does not mean that such problems are not experienced or that they may not be detrimental to women’s HRQOL. The findings from Zeng et al’s study showed that some Chinese survivors of cervical cancer defined the importance of their sex life and the harmony of the sexual relationship with their husband as one of the major indicators of their quality of life [29]. In a study undertaken in Korea, anxiety about sexual performance was prevalent in cervical cancer survivors and dyspareunia was a major and distressing problem for women who received radiotherapy for cervical cancer [30]. Although Chinese culture obscures understanding of patients’ true sexual concerns, professionals are expected to clarify the problems in this respect and find effective solutions. Thus how to improve Chinese women’s acceptability of the sex-related items within the subscale of the CCS is an important and challenging task. Additional work is required.

Although the personal interviews revealed some important clues about the properties of the instrument, given that the intent is to use this translation for measurement purposes, the psychometric tests provide evidence of the reliability and validity of the scale.

Essentially, internal consistency represents the average of the correlations among all the items in the measure. This implies that each item should correlate with the subscale score [17]. The item-subscale correlations for all the items were acceptable except for item C6 (I have concerns about my ability to have children) in the CCS. Consistent with the results from the personal interviews, the item-subscale correlation for item C6 was lower than 0.2. It did not meet the acceptability requirement and seemingly should be omitted from the scale when used in a Chinese context [16,17]. The alpha coefficient of the CCS without item C6 was actually a little higher than that of the CCS. However, 93.3% of the subjects already had children. They tended to have the same, or a similar, response to the item as in the personal interviews. There is a possible source of bias for the item, contributing to the low item-subscale correlation of item C6. Further investigation in a more representative sample including a sufficient number of childless women with cervical cancer is required to clarify the item’s adequacy in a Chinese cultural context.

The Cronbach’s alpha coefficient of the FACT-Cx was 0.88 and all the subscales were higher than 0.70 except for the CCS which was 0.57. This indicated that the FACT-Cx had good reliability whereas the CCS’s reliability was marginal. Likewise, other studies also found a relatively low Cronbach’s alpha coefficient for some cancer-site-specific subscales, such as the lung-cancer-specific subscale and breast-cancer-specific subscale [5,13,14]. The lung-cancer-specific subscale was 0.68 in a western cultural context in Cella et al’s investigation [5] and 0.56 in a Chinese cultural context in Wan et al’s study [13]. The breast-cancer-specific subscale was only 0.59 in a Chinese sample [14]. One possible explanation for this may be that these subscales usually cover diverse aspects related to treatment, complications, side effects, and particular concerns, with the result that the items are not really related [13]. The relative homogeneity of the sample probably also accounted for the low alpha value. Reliability involves the ratio of variability among subjects to total variability, thus conducting a test study on an extremely heterogeneous sample ensures good reliability of an instrument [17]. Compared to the whole population of women with cervical cancer, the sample in this study was perhaps too homogeneous. Because the subjects were recruited from two top-grade university hospitals located in a big city in China, they tended to have higher education and higher income than the average. Furthermore the majority of the subjects were in an early stage of their disease. If more patients in the late stages were included and their financial status varied considerably, the internal consistency would probably improve.

The results of the factor analysis paralleled the original with two minor discrepancies. Item P6 “I feel ill” loaded on the EWB factor rather than on the PWB factor. From Chinese people’s perspectives, “someone feels ill” does not mean “someone is really ill” and rather reflects his/her feelings for his/her body. The item is thus highly relevant to EWB, an intuitively more attractive location for this item. Item E2 “I am satisfied with how I am coping with my illness” in the EWB subscale cross-loaded on the PWB, SFWB and FWB factors at similar factor loadings. The explanation may lie in that the extent to which a patient is satisfied with her coping with disease is largely determined by the consequences of her coping, as reflected in physical, social/family and functional wellbeing. If things go well in these three areas, the patient interprets it as the result of her successful coping and thereby feels satisfied.

A reliable and valid HRQOL instrument should be sensitive to clinical subgroups’ differences. It was hypothesized that the HRQOL questionnaire had the ability to differentiate between patients under active treatment and those who were not, patients with different levels of performance status, and patients at different stages of their disease. As anticipated, there was a significant difference in patients’ FACT-Cx scores between treatment and non-treatment groups, as well as in FACT-G, PWB and CCS scores. Furthermore, significant differences in the PWB, FWB, CCS, FACT-G and FACT-Cx, were also found among the five groups categorized by level of performance status. The results show that known-group validity is good in Chinese cultural context, consistent with the results in US women with cervical cancer [15]. That implies that the FACT-Cx has adequate known-group validity cross-culturally. However, there was no significant difference in HRQOL among the patients at different cancer stages, except for EWB. One possible explanation, as mentioned above is that the sample was too homogeneous. The majority of subjects were at an early stage of their cancer and the differences in HRQOL among the groups at different stages were too small to be detected.

There are some limitations which need to be recognized. First of all, it is the representativeness of the sample. Provided that all the subjects were recruited from the two top-level hospitals in a big city, they tended to be better off in terms of socio-economic aspects. In addition the majority of the subjects were in an early stage of their disease. This may limit the generalizability of the findings. Secondly, the presence of the investigator may have improved the quality of responses, however it may also have influenced the patients’ choices, especially for some sensitive items, such as the sex-related items. There may also have been an effect from social desirability.

## Conclusion

From the viewpoint of Chinese women with cervical cancer, the Chinese version of the FACT-Cx covered the major areas that were perceived as important. It was regarded as easy to understand and administer. In general, its internal consistency was acceptable. The Chinese version of the FACT-G demonstrates a factor structure comparable with the original, and the FACT-Cx and CCS showed acceptable known-group validity. Thus the FACT-Cx appears suitable to serve as an instrument for measuring HRQOL in women with cervical cancer in Mainland China. However, some limitations existed in the CCS subscale. Firstly, the internal consistency was marginal with one item that of loss of reproductive ability having low item-subscale correlation. The qualitative and quantitative findings both suggest further research to improve the understanding and measurement of Chinese survivors’ concerns about loss of reproductive ability. In addition, due to Chinese people’s view of sex as a taboo topic, improving the response rate for sex-related items in the CCS is another challenge for Chinese investigators. The homogeneity and under-representativeness of our sample undermined the reliability and generalization of the findings. Further investigation of the scale’s psychometric properties is recommended in more heterogeneous samples.

## Competing interests

No conflict of interest is known.

## Authors’ contributions

The first author was responsible for planning, conducting the study and drafting the manuscript. All authors were involved in planning the study and amending the manuscript. All authors read and approved the final manuscript.
